# TRanscutaneous lImb reCovEry Post-Stroke (TRICEPS): study protocol for a randomised, controlled, multiarm, multistage adaptive design trial

**DOI:** 10.1136/bmjopen-2024-092520

**Published:** 2025-03-26

**Authors:** Sheharyar S Baig, Cara Mooney, Kirsty McKendrick, Kate E M Duffy, Ali N Ali, Jessica N Redgrave, Esther Herbert, Simon Waterhouse, Li Su, Avril Drummond, Jesse Dawson, Munyaradzi Dimairo, Katie Biggs, Cindy Cooper, Arshad Majid

**Affiliations:** 1 Sheffield Institute for Translational Neuroscience, The University of Sheffield, Sheffield, UK; 2 School of Medicine and Population Health, The University of Sheffield, Sheffield, UK; 3 Department of Psychiatry, University of Cambridge, Cambridge, UK; 4 School of Health Sciences, University of Nottingham, Nottingham, UK; 5 School of Cardiovascular and Metabolic Health, University of Glasgow, Glasgow, UK

**Keywords:** Stroke, Rehabilitation medicine, Clinical trials

## Abstract

**Introduction:**

Arm weakness after stroke is one of the leading causes of adult-onset disability. Invasive vagus nerve stimulation (VNS) paired with rehabilitation has been shown to improve arm recovery in chronic stroke. Small studies of non-invasive or transcutaneous VNS (tVNS) suggest it is safe and tolerable. However, it is not known whether tVNS paired with rehabilitation is effective in promoting arm recovery in chronic stroke and what the mechanisms of action are.

**Methods and analysis:**

TRICEPS is a UK multicentre, double-blinded, superiority, parallel-group, three-arm two-stage with an option to select promising arm(s) at 50% accrual, individually randomised, sham-controlled trial. Up to 243 participants will be randomised (1:1:1) using minimisation via a restricted, web-based centralised system. tVNS will be delivered by a movement-activated tVNS system (TVNS Technologies), which delivers stimulation during repetitive task practice. Rehabilitation will consist of repetitive task training for 1 hour a day, 5 days per week for 12 weeks. Participants will be adults with anterior circulation ischaemic stroke between 6 months and 10 years prior with moderate-severe arm weakness. The primary outcome measure will be the change in Upper Limb Fugl-Meyer total motor score at 91 days after the start of treatment. Secondary outcome measures include the Wolf Motor Function Test, the Modified Ashworth Scale to assess spasticity in the affected arm and the Stroke-Specific Quality of Life Scale. A mechanistic substudy including 40 participants will explore the mechanisms of active versus sham tVNS using multimodal MRI and serum inflammatory cytokine levels. Participant recruitment started on 30 November 2023.

**Ethics and dissemination:**

The study has received ethical approval from the Cambridge Central Research Ethics Committee (REC reference: 22/NI/0134). Dissemination of results will be via publications in scientific journals, meetings, written reports and articles in stakeholder publications.

**Trial registration number:**

ISRCTN20221867.

**STRENGTHS AND LIMITATIONS OF THIS STUDY**

- This is, to our knowledge, the largest double-blinded, multicentre, randomised controlled trial of vagus nerve stimulation (VNS) in stroke.
- A closed-loop, movement-activated device allows the self-delivery of transcutaneous VNS at home with objective monitoring of treatment concordance.
- The trial has an efficient two-stage adaptive design. Prespecified stop-go criteria will allow continued enrolment to treatment groups where there is early promising evidence of efficacy and discontinuation of futile treatment groups.
- Participants with haemorrhagic stroke or posterior circulation ischaemic stroke are not eligible.
- This study will only address upper limb impairments poststroke.

## Introduction and rationale

Stroke is a leading cause of adult-onset disability.^1^ Up to 80% of stroke survivors experience arm weakness, and in 30%–60%, this persists at 6 months.^2^ Persistent arm weakness after stroke is a key driver of reduced independence, reduced employment and impaired quality of life (QoL).^1^ Rehabilitation is a key treatment in poststroke recovery. However, the rate and magnitude of the effect of active therapy are only partially restorative in chronic stroke (>6 months postonset).^3^ Novel strategies to increase the likelihood of arm recovery in chronic stroke are therefore a key priority for stroke survivors, clinicians and health services.

The vagus nerve provides bidirectional signalling between the brain and the body.^4^ In addition to being the output of the parasympathetic nervous system in the regulation of the heart and vasculature, it has key roles in the regulation of central and peripheral inflammation.^4^ Invasive vagus nerve stimulation (VNS) is well established as a treatment for medically refractory epilepsy.^5^ The VNS Rehabilitation (VNS-REHAB) study demonstrated that surgically implanted VNS temporally paired with rehabilitation therapy resulted in greater improvements in arm weakness than rehabilitation alone.^6^ The mechanism of this effect is likely to be cholinergic reinforcement, which increases task-specific plasticity in response to upper limb training. Several preclinical studies have shown that non-invasive transcutaneous VNS (tVNS), typically via the auricular branch of the vagus nerve (ABVN) at the cymba concha or tragus, activates vagus nerve projections centrally and improves upper limb outcomes in animal models of acute stroke.^7^ The potential advantages of tVNS over invasive VNS in stroke include the avoidance of safety risks from general anaesthetic and surgery, a lower overall cost, the ability to remove and redistribute the device from non-responders and the potential for use in the acute phase poststroke where invasive surgery is unfeasible.^8^ Pilot clinical studies have demonstrated that tVNS paired with rehabilitation can result in greater improvements in arm recovery in subacute and chronic stroke compared with sham stimulation.^9 10^ In a small unblinded pilot study, 12 participants with subacute chronic stroke completing a 6-week rehabilitation programme with concurrent tVNS had a mean 10.1-point increase in ULFM motor subscore.^11^ However, the efficacy of tVNS on arm recovery in chronic stroke and the relative effect size compared with invasive VNS is still not known. The mechanisms through which VNS may promote arm recovery in chronic stroke are not well understood, but it appears that VNS must be delivered during or immediately after limb movements to promote neuroplasticity.^4^ Furthermore, the activation of the central cholinergic pathways, specifically via the alpha-7 nicotinic acetylcholine receptors, may be a key mediator of the treatment effect.^4^ In a recent evaluation of ongoing clinical trials of tVNS in stroke, some key research gaps in tVNS and stroke have emerged.^12^ These include the absence of robust biomarkers of tVNS that can be used to evaluate the mechanism and optimise treatment dose and duration.^12^

## Study aims and objectives

The TRanscutaneous lImb reCovEry Post-Stroke (TRICEPS) trial aims to investigate the efficacy and mechanism of tVNS paired with upper limb rehabilitation on arm recovery in chronic stroke. The primary objective is to determine whether tVNS paired with self-delivered home rehabilitation therapy of the affected arm poststroke reduces arm motor impairment at 3 months from the start of treatment compared with self-delivered home rehabilitation therapy alone.

Secondary objectives are to determine: (a) whether the benefits of tVNS and self-delivered home rehabilitation therapy in reducing arm motor impairment observed at 3 months from the start of treatment are sustained; (b) whether tVNS and self-delivered home rehabilitation therapy improve other outcome measures related to sensory modalities, neurological deficit, QoL, depression, general anxiety, fatigue, pain, spasticity, strength and activities of daily living and (c) the safety of tVNS and self-delivered home rehabilitation therapy.

An imaging substudy will explore whether tVNS changes the cerebral representation of the affected arm, cerebral perfusion and functional connectivity and how they relate to reduced arm motor impairment.

## Methods

Critical details that could unblind or unmask the trial have been concealed while the trial is ongoing. A redacted version of the full protocol is available via https://fundingawards.nihr.ac.uk/award/NIHR133169. A full unredacted protocol with detailed methods will be accessible via this link when the study is completed. The core trial dataset is outlined in online supplemental table 1. A completed Standard Protocol Items: Recommendations for Interventional Trials (SPIRIT) checklist for the current manuscript is available as online supplemental appendix A.

## Trial design

**Figure 1:**
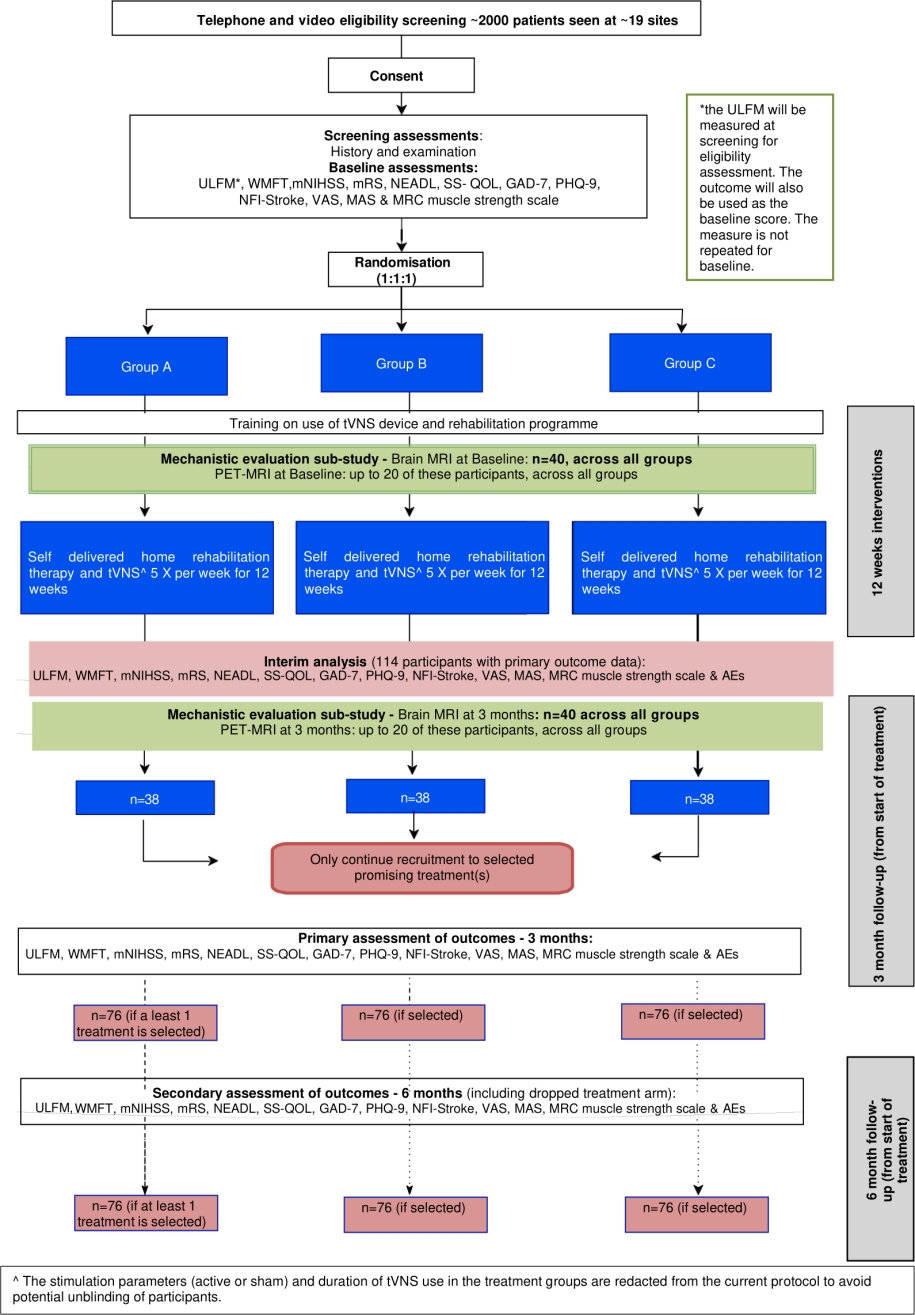
Study flow diagram. AE, adverse event; mNIHSS, Modified National Institute of Health Stroke Scale; SS-QOL, Stroke-Specific Quality-of-Life; tVNS, transcutaneous vagus nerve stimulation; ULFM, Upper Limb Fugl-Meyer; WMFT, Wolf Motor Function Test; NEADL - Nottingham Extended Activities of Daily Living; mRS - Modified Rankin Scale; GAD-7 - General Anxiety Disorder Questionnaire 7; PHQ-9 - Patient Health Questionnaire-9; NFI-Stroke - Neurological Fatigue Index for Stroke; MAS - Modified Ashworth Scale; MRC - Medical Research Council; PET - Positron Emission Tomography.

TRICEPS is a multicentre, double-blinded, parallel-group, superiority, individually randomised, sham-controlled, three-arm, two-stage adaptive trial. This includes an internal pilot with a stop-go decision following 6 months of recruitment to assess feasibility (see Section 8.4 of the full protocol) and an interim analysis after the first stage to allow for the selection of promising treatment groups in the second stage as well as reassessing dropout rate and adjusting the sample size accordingly. Early trial stopping for efficacy has not been factored into the design. The flow diagram of the study design and participant interventions through the trial is summarised in figure 1.

### Patient population

Adults with anterior circulation ischaemic stroke between 6 months and 10 years previously with upper limb weakness.

### Inclusion and exclusion criteria

#### Inclusion criteria

- Aged 18 years or greater.
- Anterior circulation ischaemic stroke (internal carotid, middle cerebral artery, anterior cerebral artery or anterior circulation lacunar stroke) between 6 months and 10 years previously.
- Baseline Upper Limb Fugl-Meyer (ULFM) total motor score of 20–50 (inclusive) indicating moderate to severe arm motor impairment.
- At least 10 degrees of active wrist extension, 10 degrees of active thumb abduction/extension and 10 degrees of active extension in at least two additional digits in the affected arm.
- Able to participate in active rehabilitation therapy, provide feedback on adverse events (AEs) and give informed consent based on clinical judgement.

#### Exclusion criteria

- Has significant other impairment of the upper limb, for example, a frozen shoulder.
- Has severe spasticity (eg, as identified by the ULFM).
- Has other health conditions that prevent engagement with rehabilitation therapy, for example, advanced dementia.
- Has severe aphasia and either: (a) informed consent is unlikely based on the Consent Support Tool, (b) engagement with repetitive task training is difficult or (c) inability to communicate AEs from tVNS.
- Currently participating in another interventional stroke rehabilitation trial.
- Pregnant or trying to get pregnant.
- Has a pacemaker or another implanted electrical device.
- Has a cochlear implant or other similar device.
- Is currently receiving therapy or treatment to reduce arm motor impairment and would not be willing to stop for the duration of the trial.
- Has previously experienced a haemorrhagic stroke.
- Administration of botulinum toxin in the affected arm within the previous 3 months (participants must also be willing to not have further injections for the duration of the trial).

The entry criteria of a ULFM motor subscore of 20–50 is in line with the VNS-REHAB trial.^6^ This will enable a more direct comparison of the relative efficacy of invasive versus non-invasive VNS. The study team, including a patient and public involvement panel, felt that participants scoring <20 points on the ULFM may struggle to carry out repetitive task practice, thereby limiting the potential for meaningful gains, and those scoring >50 points on the ULFM have less impairment, and thereby the score may have a ceiling effect. The range of 20–50 aims to be as inclusive as possible while acknowledging the possibility of different trajectories of rehabilitation response according to stroke severity.^13^ The inclusion of participants from 6 months to 10 years poststroke is justified by previous work on intensive upper limb rehabilitation and was codesigned in consultation with stroke survivors. Spontaneous biological recovery is a prominent mechanism of recovery in the first 3–6 months poststroke, followed by a more stable chronic deficit.^14^ In the chronic phase (after 6 months), improvements in limb function are driven by rehabilitation, often delivered at high doses.^15 16^ Previous studies of intensive upper limb rehabilitation in stroke have shown improvements even several years poststroke onset.^16^

### Identification and recruitment

Potential participants will be identified through several possible routes. First, individual community stroke rehabilitation teams will be asked to identify participants under their active or historical care who meet the eligibility criteria. Second, individual sites will be asked to review records for potentially eligible stroke survivors via the Sentinel Stroke National Audit Programme database (routinely collected national audit record). Third, the trial will be advertised electronically via the trial website and social media advertisements and physically via posters in community rehabilitation and care settings with contact details of their local site and/or the central research team. Potential participants will be provided with an invitation letter and participant information sheet (online supplemental appendix B) via email or post. A telephone screening checklist outlining the core inclusion and exclusion criteria will be completed with the participant. A video screening assessment will be conducted where possible to determine the range of arm weakness and determine likely eligibility. Participants will be invited to an enrolment visit where they provide written informed consent for trial participation before completing the ULFM assessment. The consent form is shown in the online supplemental appendix C. If they meet the inclusion criteria, they will complete the remainder of the outcome assessments (as below) and proceed to randomisation; sites and participants will have the option to divide the baseline visit into two appointments if required. Participants who are eligible for the mechanistic substudy will be given an information sheet (online supplemental appendix D), and written informed consent (online supplemental appendix E) for participation will be required for participation in the substudy. Recruitment to the TRICEPS trial began on 30 November 2023.

### Randomisation

Eligible and consenting participants will be randomised using a centralised, validated, restricted, web-based randomisation system (SCRAM - Sheffield Clinical Trials Research Unit Randomisation System) hosted by the University of Sheffield Clinical Trials Research Unit (CTRU). Participants will be randomised 1:1:1 to one of three treatment groups using minimisation with a masked random probabilistic element to reduce the predictability of treatment allocation. A prespecified number of initial participants will be randomised using simple randomisation after which the randomisation will be minimised according to age (≤60, >60 years), baseline ULFM score (20–35, 36–50) and biological sex at birth (male, female) and by recruiting site to achieve good balance across treatment groups with respect to potential prognostic factors. The allocation ratio will be updated to 1:1, and the minimisation algorithm will be updated if a treatment is dropped at interim analysis.

### Blinding

Device set-up, device training and the therapy intervention will be delivered by unblinded team members. Depending on the site, only the research therapist (or research nurse) who will train participants on the use of the tVNS device will be unblinded. The CTRU data managers, trial manager and research assistant will also be unblinded to aid in operational aspects of the trial. All outcome assessments will be completed by a blinded trial outcomes assessor. TRICEPS uses a sham control device which will be the same as the active tVNS device, but the stimulation will be set at the minimum stimulation intensity level. Participants will be informed that they ‘may or may not’ be able to feel the stimulation from the device and that this varies between individuals. The research therapist or nurse will be provided with a script to guide this conversation and ensure all participants are given the same information to help maintain blinding. Emergency unblinding in case of a medical emergency or serious AE (SAE) will be permissible on an individual basis.

Participant-facing documents will be designed in such a way that potential participants receive enough information to be able to make an informed decision about participation but will not disclose too much about the difference between the treatment groups in order to preserve blinding. At the end of the 6-month intervention, participants will be asked whether they thought they were in the active or sham intervention; these results will be reported as an indicator of the blinding process.

Sheffield CTRU statisticians independent of the conduct of this trial will access unblinded data for monitoring purposes and to conduct interim analysis. They will communicate unblinded results to the DMEC. Trial statisticians will be blinded throughout the trial.

### Intervention

Participants will be randomised 1:1:1 to one of the treatment groups:

- Group A: a treatment group that receives tVNS* with self-delivered rehabilitation (five times a week) for 12 weeks.
- Group B: a treatment group that receives tVNS* with self-delivered rehabilitation (five times a week) for 12 weeks.
- Group C: a treatment group that receives tVNS* with self-delivered rehabilitation (five times a week) for 12 weeks.

*The stimulation parameters (active or sham) and duration of tVNS use in the treatment groups are redacted from the current protocol to avoid potential unblinding of participants.

### tVNS device

**Figure 2:**
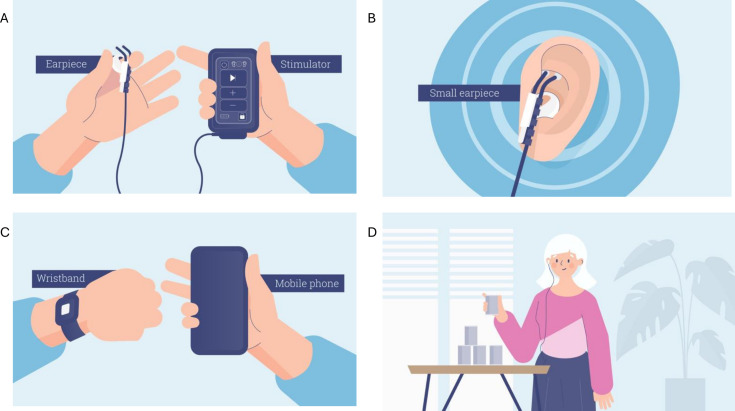
tVNS system. (A) tVNS stimulator; (B) tVNS stimulation site at cymba concha; (C) smart wristband and mobile phone; (D) tVNS delivery during rehabilitation. Figures created by Morph. tVNS, transcutaneous vagus nerve stimulation.

All treatment groups will be given a wristband with a movement sensor (mbientlab), mobile phone and a tVNS stimulator with earpiece (tVNS Technologies). Participants will undergo device training at their first therapy visit, which will be supplemented with written and video-based training materials. Participants will wear the smart wristband during their therapy sessions, which will detect limb movement and trigger a pulse of tVNS via a preinstalled application on the provided mobile phone; this will enable the pairing of limb movement to stimulation (figure 2). The device will also record how long the device has been used and the number of motion-triggered stimulations.

The stimulator delivers tVNS to the ABVN at the cymba concha at 25 Hz and 0.1 ms pulse width. The intensity for group A will be set at 0.1 mA while for groups B and C, this will be set at a variable range between the individual participant’s minimum perceived threshold (minimum, at least 0.5 mA) and pain threshold (maximum, up to 5 mA). The mobile device provided will monitor adherence to intervention protocols. Participants will be able to modulate their intensity according to their tolerability on a day-to-day basis, but the device will not display the absolute intensity of the current. Negligible stimulation in the placebo group was chosen in order to maintain the functionality of the device and to minimise unblinding of participants.

### Rehabilitation

Each participant will be trained by a physiotherapist or occupational therapist to self-deliver therapy at home. Training will take place face-to-face in a clinical setting. The therapy will be tailored to the participant’s own set of functional impairments and based on the principles of repetitive task training.^17^ The aim is for approximately 7–10 tasks (such as turning cards, moving objects and opening/closing bottles) with 30–50 repetitions per task within a 1hour- session. A delegated unblinded research team member will contact the participant at 2-week intervals during the treatment period (12 weeks). The purpose of this contact is twofold; first, to identify any issues with rehabilitation therapy or the device; second, to progress the therapy as required.

### Primary and secondary outcome measures

Assessments and schedule of procedures are outlined in the online supplemental table 2 and figure 1. The primary outcome is the change in ULFM total motor score at 3 months from baseline (start of treatment). The ULFM total motor score consists of 33 items, each scored on a three-point ordinal scale (0, 1 or 2) with a range of 0–66;^18^ lower scores are associated with impaired function. All research staff carrying out the ULFM assessment will be trained and assessed with an accredited online programme. Further assessments that form the basis of secondary outcome measures are listed in full in online supplemental table 2 (schedule of procedures). These include the other components of the ULFM assessment (sensation, passive joint motion and joint pain), the Wolf Motor Function Test (WMFT),^19^ the Modified National Institute of Health Stroke Scale (mNIHSS)^20^ and the Stroke-Specific Quality-of-Life (SS-QOL) Scale.^21^ Training in the Wolf Motor Function Test will be carried out with video materials provided by the central team. Complete details of secondary outcomes are outlined in Section 8.2 of the full protocol.

### Trial monitoring and oversight

The day-to-day management of the trial will be supervised by the trial management group (TMG) and will meet at least monthly. The conduct of the trial will be overseen by the trial steering committee (TSC) and data monitoring and ethics committee (DMEC). The DMEC will meet regularly and review reports (blinded and unblinded) provided by the Sheffield CTRU to assess the trial progress, safety data and efficacy data to make recommendations on trial adaptations. They will advise the TSC who will also meet regularly to monitor the trial, review blinded data and act on DMEC recommendations on behalf of the funder and sponsor.

### Sample size justification and interim analysis

Rehabilitation alone has been shown to improve the ULFM total motor score by around 3 points^17^; an improvement of 6 points is considered the minimal clinically important difference (MCID).^22^ The active treatment group would need to improve the ULFM by 3 points more than sham tVNS and rehabilitation to be clinically meaningful. The trial will require 228 participants in total (76 per group) with ULFM outcome at 3 months. An interim analysis for treatment selection will be performed when 114 participants in total (38 per group) have accrued ULFM outcome at 3 months. This assumes a 1:1:1 allocation ratio, 85% marginal power and 2.5% one-sided family-wise type I error. We expect a 6% dropout rate,^16^ so the initial sample size is inflated to 243 participants (81 per group). However, this dropout rate will be re-estimated at an interim analysis, and the sample size adjusted accordingly to achieve 228 participants with primary outcome data. We chose a treatment selection rule to minimise the risks of dropping potentially effective tVNS treatments or selecting ineffective treatments at an interim analysis. A tVNS treatment will be selected to progress to stage 2 if the test statistic is >0.792 (corresponding to a 1-point ULFM mean difference in change compared with a shared control arm; 0.18 of the expected SD of 5.5^11^ and one-third of our targeted MCID). Such a small effect observed halfway through the trial will be very unlikely to improve close to or beyond our targeted MCID even if the treatment is carried forward until the end. Thus, we will drop a tVNS treatment early if it fails to demonstrate an effect of>1/3 of the MCID. At the end of stage 2, a treatment will be declared efficacious if the test statistic is above 2.176—chosen to guarantee that the chance of incorrectly rejecting at least one null hypothesis is controlled at 2.5% (one-sided tests).^23^ See Section 11.1 of the protocol for details on sample size with rationale and aspects of interim analysis including adaptation decision rules with rationale and operating characteristics of the adaptive design.

### Statistical analysis

Details of all statistical analysis methods relating to the interim and final (stage 2) analyses will be documented in an open-access and prespecified statistical analysis plan before accessing unblinded data. Interim and final (stage 2) analyses for efficacy will be based on a modified intention-to-treat population—all randomised participants who have outcome data regardless of non-compliance, protocol deviations or withdrawals. Sensitivity analysis will be performed on the per-protocol population—all randomised participants who adhered to treatment with no major protocol deviations. Adherence is defined as completing 40/60 therapy sessions for at least 30 min per session (all groups) and completing at least 1 hour of tVNS with activities of daily living (ADLs). Safety population will include eligible participants with informed consent, and analysis will be based on the treatment they received. Reporting will adhere to the Adaptive Designs Consolidated Standards of Reporting Trials Extension.^24^

### Primary outcome

The primary outcome (change in ULFM total motor score at 3 months) will be analysed using a mixed effects linear regression model adjusted for fixed effects covariates (baseline ULFM score, age AND sex) and site (random effect). Treatment effects will be presented as the adjusted mean difference in change with multiplicity-adjusted CIs and associated p values. Subgroup analyses will be undertaken to explore the effect of important variables related to the participant and their treatment on the primary outcome. These subgroups are:

- Age (≤6, >60 years).
- Baseline ULFM score (20–35, 36–50).
- Biological sex at birth (male, female).
- Side of hemiparesis (left, right).
- Time since stroke (<12 months, >12 months).
- Baseline mNIHSS score (0–4=minor, 5–20=moderate and 21–31=severe).

### Secondary outcomes

The continuous secondary outcome measures at 3 and 6 months will be analysed with the same mixed effects linear regression model; the adjusted mean difference in change in these outcomes will be presented with 95% CIs with no adjustment for multiple testing. Ordinal outcomes will be analysed using an ordinal logistic regression model to assess the shift in the distribution of scores at follow-up that is attributed to treatment. The proportion of participants achieving at least 6 points improvement in ULFM total motor score (at 3 and 6 months) compared with baseline will be analysed using a mixed effects logistic regression model adjusted for fixed covariates (baseline ULFM scores, age and sex) and site (random effect). Adjusted difference in proportions between each tVNS compared with the control, with associated 95% CIs, will be postestimated via margins.

### Safety monitoring and reporting

AEs and SAEs are defined as events that occur between randomisation and completion of their 6-month follow-up. These may be identified by the participant, research nurse, therapist or any other individual at any point in the trial. AEs will be recorded on the AE report form within the participant CRF. Some expected AEs include those known to be associated with tVNS and/or rehabilitation, including skin irritation, headache, nausea and limb pain.^25^ In a recent systematic review of auricular tVNS, no adverse cardiac events were reported in 484 212 min-days of stimulation.^26^ SAEs will be reported to the CTRU immediately and within one working day from identification. Unexpected SAEs related to the intervention will be reported to the research ethics committee within 15 working days. AEs and SAEs will be reviewed by the DMEC and TSC regularly.

### Mechanistic substudy

Participants willing to travel to the central site (Sheffield) and with no contraindications for an MRI scan will be invited to participate in the mechanistic substudy and provide written informed consent. The aim of the substudy is to establish whether active tVNS paired with rehabilitation enhances cortical plasticity, cerebral blood flow and metrics of brain oxygen metabolic profiles or reduces systemic inflammation compared with sham tVNS with rehabilitation therapy. It will also explore whether this is associated with improvement in upper limb function and whether baseline features of the MRI such as lesion size, location or the presence of white matter ischaemic changes are predictors of the response to active tVNS. Up to 40 participants will have a 60-min multimodal MRI scan (T1, T2, Diffusion Tensor Imaging (DTI), Fluid Attentuated Inversion Recovery (FLAIR), Arterial Spin Labeling (ASL), task and resting state functional MRI (fMRI)) at baseline (within 4 weeks of baseline measure assessments and prior to commencing the tVNS/therapy programme) and at 3 months. Up to 20 of these participants will also have an 18F-fluorodeoxyglucose positron emission tomography (18F-FDG-PET) scan concurrent to the multimodal MRI. Briefly, the task fMRI paradigm consists of a block design with 3×30 s blocks of active flexion-extension movements of either the affected or unaffected fingers, wrist or elbow at 1 Hz (six movement tasks) interspersed with 30 s rest blocks. Preprocessing of the functional MRI results will include an analysis pipeline of stroke lesion segmentation, motion correction, slice timing correction, coregistration to structural MRI, normalisation to a shared template and smoothing with a Gaussian kernel. A general linear model will be applied to identify task-related activation compared with the rest. First-level individual participant contrasts will be generated, and second-level analysis will compare activations postintervention versus preintervention in active versus sham treatment groups. Correction for multiple comparisons will be applied via family-wise error or false discovery rate correction. Region of interest analysis will be carried out in relevant sensorimotor areas including the primary motor cortices, supplementary motor areas, premotor cortex and primary somatosensory cortex. Analysis will be carried out in the latest version of statistical parametric mapping.^27^ Up to 40 participants will have blood samples collected at baseline and 3 months for serum analysis of systemic inflammatory cytokines including interleukin 1 alpha, tumour necrosis factor alpha and interferon gamma. Blood samples will be centrifuged and the supernatant stored at −70°C for future analysis.

### Ethics and dissemination

This study has received ethical approval from the Cambridge Central Research Ethics Committee (12/10/2022, REC reference: 22/NI/0134) and is registered as a clinical trial (ISRCTN20221867). The study sponsor is Sheffield Teaching Hospitals National Health Service Foundation Trust. The trial will be conducted in accordance with this protocol and the International Council for Harmonisation's Good Clinical Practice guideline. Important protocol modifications (eg, changes to eligibility criteria, outcomes and analyses) will be communicated to relevant parties including oversight committees, funders, investigators, Research Ethics Committe (REC), Health Research Authority (HRA) and trial registries.

Data confidentiality as per the Data Protection Act 2018 will be followed. Trial participants will be provided with a unique study ID number, and all relevant data will be entered into the password-protected and restricted Sheffield CTRU in-house electronic data management system. Access will be granted to authorised representatives from the sponsor, host institution and regulatory authorities for audit and monitoring purposes.

Any significant modifications to the protocol, for example, those that impact study procedures, study design, eligibility criteria or participant safety will require a formal amendment to the protocol. This will be agreed on by the TSC, funder and ethics committee before implementation and notification of health authorities.

The results of the clinical trial will be disseminated through peer-reviewed scientific journals, at clinical and academic medical and rehabilitation conferences and to trial participants and patient groups. A summary of the research will be made available on the University of Sheffield CTRU website.

Requests for patient-level data and statistical code should be made to the corresponding author and will be considered by members of the original TMG, including the chief investigator and members of CTRU, who will release data on a case-by-case basis. Data will be shared following the principles for sharing patient-level data as described by Smith *et al* (2015). The data will not contain any direct identifiers; we will minimise indirect identifiers and remove free-text data to minimise the risk of identification.

## supplementary material

10.1136/bmjopen-2024-092520 online supplemental file 1

10.1136/bmjopen-2024-092520 online supplemental file 2

10.1136/bmjopen-2024-092520 online supplemental file 3

10.1136/bmjopen-2024-092520 online supplemental file 4

10.1136/bmjopen-2024-092520 online supplemental file 5

10.1136/bmjopen-2024-092520 online supplemental file 6

10.1136/bmjopen-2024-092520 online supplemental file 7

## Data Availability

Data are available upon reasonable request.
