# Supplementary material for: TRanscutaneous lImb reCovEry Post-Stroke (TRICEPS): study protocol for a randomised, controlled, multiarm, multistage adaptive design trial
Source: BMJ Open. 2025 Mar 26;15(3):e092520. doi: 10.1136/bmjopen-2024-092520 (PMC11950934; doi:10.1136/bmjopen-2024-092520)
Supplement: online supplemental file 2 [file bmjopen-15-3-s002.docx]

**TRICEPS Participant Information Sheet**

Study Title: **TR**anscutaneous l**I**mb re**C**ov**E**ry **P**ost**-S**troke **(TRICEPS)**

*You are invited to take part in a research project to find out whether stimulation of a nerve supplying the brain (the vagus nerve), when combined with rehabilitation therapy, improves arm function after a stroke.*

*Before you decide whether to take part, it is important that you understand why the research is being done and what it will involve. Please take time to read the following information sheet carefully and discuss it with friends, or relatives, if you wish.*

*Please ask us if there is anything that is not clear to you or if you would like more information.*

# What is the purpose of this trial?

This trial aims to find out whether Transcutaneous Vagus Nerve Stimulation (TVNS) improves arm function after stroke when it is used alongside rehabilitation therapy over 12 weeks. Many people have long term problems with their arm after stroke. It was recently shown that stimulating the vagus nerve during rehabilitation sessions led to better recovery of the arm than rehabilitation alone. That trial used an implanted stimulator, which required surgery, and the rehabilitation therapy was mostly delivered in hospital.

In this study we want to see if we can improve arm recovery by using a non-invasive stimulator which clips on to the ear and with the rehabilitation delivered at home. ‘Non-invasive’ means surgery is not needed. We want to assess this in the most thorough way so we will compare non-invasive vagus nerve stimulation with a sham that produces very low stimulation, like a placebo. You will not know which version you receive.

**Why have I been invited to take part in the trial?**

You have been invited as you may have arm weakness following a stroke that occurred between 6 months and 10 years ago. You may have responded to one of our advertisements or you were identified by your clinical team.

**Do I have to take part?**

No, you do not have to take part; it is up to you to decide. We will describe the trial throughout this information sheet, which you can keep. You do not have to decide today, you can take the time to read this information sheet and contact the research team using the details provided at the bottom of this sheet if you are interested. The clinical research team might also contact you to see if you are interested in taking part or have any questions. If you do agree to take part, you are free to withdraw (stop) at any time, without giving a reason. Your decision to take part will not affect the standard of care you receive.

# What would taking part involve?

The trial involves wearing a TVNS earpiece and wristband whilst doing rehabilitation therapy at home. Some participants will also be asked to wear the device while performing their usual daily activities. You will be given specific instructions regarding the device and the rehabilitation exercises.

A summary of what the trial involves is shown in the table below and is described next.

Telephone screening

If you are interested in taking part in the trial, you will be asked some initial questions over the phone to make sure you are eligible to take part.

Face-to-face consent, screening, and treatment group randomisation

If you are potentially eligible to take part, you will then be invited to a face-to-face enrolment appointment to confirm this. If you are eligible, you will be asked to complete a consent form to confirm that you are happy to take part.

We will then do some checks to confirm that you are eligible. This will involve a member of the research team going through screening assessments with you. These include a review of your medical history and physical examination. This is to make sure it is safe for you to take part.

Please note that the device should not be used on persons who are pregnant. If you are of child bearing age then you will be asked to carry out a pregnancy test to confirm you are not pregnant at the face to face visit.

Site staff will then go through some assessments and questionnaires with you. This will include a physical assessment to assess motor functioning, sensation and joint functioning. This is called the Upper Limb Fugl Meyer (ULFM) assessment.

If these checks confirm that you are eligible, you will then be randomly assigned to a treatment group (in the same appointment). You will not be told which treatment group you are in. The only research staff that will know which treatment you are receiving is a member of the clinical research team who shows you how to use the device. This is so knowledge of the treatment you are receiving does not consciously or subconsciously cause a change in your behaviour, as a participant, or the behaviour of investigators, which can bias trial results. You will have the option of being told what treatment you received after the trial has finished. The treating therapist will draw up a personalised therapy plan for you. You will be trained on how to self-deliver the therapy and use the TVNS device.

This appointment will take place in a clinical setting, such as a stroke centre or hospital and will last approximately 2-2½ hours.

The 12-week TVNS and rehabilitation period

You will complete self-delivered rehabilitation therapy whilst wearing the device provided by the research team at home. All participants will do this, and you will undertake the self-delivered therapy while wearing the device for 1 hour, 5 times a week. Some people will, **in addition**, be asked to wear the device whilst doing their usual daily activities. If you are asked to do this, you will complete this on the same days you do your rehabilitation therapy. You will be asked to wear it for at least 1 additional hour and for *up to* 8 hours (you can choose between 1-8 hours).

The treatment period will last for 12 weeks and will be completed at home. Your rehabilitation therapy plan will be tailored to you. Your therapy will include repetitive tasks such as turning cards, moving objects, opening, and closing bottles.

The TVNS equipment includes a stimulator device, earpiece (called an electrode), mobile phone, charger, and a wristband (which contains a

movement sensor). You will be shown how to use the device and how it should be charged. Please note, you should not use the mobile phone for any other activities outside of the trial. You will return the mobile phone at the end of your treatment period. Data collected from the mobile phone (motion and how long the device has been worn for) will be in accordance with **“Will my data be kept confidential?”** All data will be wiped once you return your TVNS equipment. Figures 1 and 2 show what the device will look like. You will return all equipment once the 12-week treatment period has finished. If required we may arrange for a courier to collect the device from your home. Your address will be provided to the central research team for this purpose.

**Figure 1 and 2. The TVNS ear clip and stimulator**

**Image redacted for online publication**

A member of the clinical research team will organise phone or video calls with you throughout your 12-week treatment period. If you would prefer, these may be completed face-to-face at the research facility. These calls/visits will focus on your rehabilitation therapy, if there are any issues with your TVNS device and to check if you are experiencing any side effects from the treatment (please see **“what are the possible disadvantages of taking part”**)**.** If there are any issues, you may be asked to attend an additional face-to-face appointment to fix these.

A member of the central research team at The University of Sheffield may also complete some of these calls with you. The number of calls/visits will vary. On average you will have around 5 calls/visits. We will ask you to bring the equipment to any face to face visits to check how often it is being used.

You may also be invited back to your recruiting centre, or asked if you would prefer a home visit, to check the device is working as it should.

You can also contact your local research team directly via the contact details at the end of this information sheet if you are having any problems using the device or completing your therapy.

Follow-up assessments

Following the treatment period, you will attend a face-to-face appointment where a member of the research team will repeat similar assessments that were completed at your first appointment as shown in Table 1. You will also return the device at this appointment. You will then be invited to another face-to-face appointment for the same measures to be repeated, 6 months after starting treatment. These appointments will take place in a clinical setting such as a stroke centre or hospital and will last approximately 1-2 hours each.

These appointments are an important part of the trial as it allows us to assess the outcome of the TVNS and rehabilitation therapy treatment. Each person attending these appointments helps us to answer our research question. Please see Table 1 below for a summary of all measures that are involved in the trial.

**Table 1. Summary of participant assessments and questionnaires**

|  | | **Face-to-face enrolment appointment** | **3 months after treatment start date (face-to-face)** | **6 months after treatment start date (face-to-face)** |
| --- | --- | --- | --- | --- |
| **Physical assessments**  *(Approximately 1 hour and 45 minutes to complete all assessments)* | Medical history  (to check eligibility) | **⚫** |  |  |
|  | Physical examination  (to check eligibility) | **⚫** |  |  |
|  | Upper Limb Fugl Meyer (ULFM)  (to check eligibility) | **⚫** | **⚫** | **⚫** |
|  | Wolf Motor Function Test (WMFT) | **⚫** | **⚫** | **⚫** |
|  | Modified National Institute of Health Stroke Scale (mNIHSS) | **⚫** | **⚫** | **⚫** |
|  | The Modified Ashworth Scale (MAS) | **⚫** | **⚫** | **⚫** |
|  | The Medical Research Council Muscle Strength Scale (MRC Muscle Strength Scale) | **⚫** | **⚫** | **⚫** |
| **Questionnaires**  *(Approximately 25/30 minutes to complete all questionnaires)* | Modified Rankin Scale (mRS) | **⚫** | **⚫** | **⚫** |
|  | Nottingham Extended Activities of Daily Living (NEADL) scale | **⚫** | **⚫** | **⚫** |
|  | Stroke-Specific Quality-of-Life (SS-QOL) | **⚫** | **⚫** | **⚫** |
|  | Generalised Anxiety Disorder Assessment (GAD-7) | **⚫** | **⚫** | **⚫** |
|  | Patient Health Questionnaire (PHQ-9) | **⚫** | **⚫** | **⚫** |
|  | Neurological fatigue Index for Stroke (NFI-Stroke) | **⚫** | **⚫** | **⚫** |
|  | Visual Analogoue Scale (VAS) | **⚫** | **⚫** | **⚫** |

# What are the possible benefits of taking part?

You will be given a tailored 12-week rehabilitation therapy programme to do at home and a TVNS device to use for this period. This may be of value to some

participants who may not be receiving any other therapy for their arm weakness.

# What are the possible disadvantages and risks of taking part?

The TVNS device is usually well tolerated but previous studies have found some side effects. These are mild skin irritation (in 15% of cases), headaches (less than 5% of cases), dizziness, sore throat and nausea (all in less than 2% of cases).

The nerve that is stimulated in this trial can affect the heart rhythm but there has been lots of research using this device in humans with no concerns about safety of the participants.

Some participants may find participation in the trial time consuming as you will be required to attend at least three face-to-face appointments, in addition to the 12-week treatment period completed at home.

**What will happen if I do not want to carry on with the trial?**

You can withdraw from the trial at any time without giving any reason. We will keep your data up until the point that you withdraw, and we will not collect any new information from you.

**What if new information becomes available?**

Sometimes during the course of a research trial, new information becomes available about the intervention that is being studied. If this happens, the research team will inform you and discuss with you whether you want to continue in the trial. If you decide to continue in the trial, you will be asked to sign an updated consent form.

In some circumstances, on receiving new information the researcher might consider it to be in your best interests to withdraw you from the trial. The researcher will explain the reasons.

We will also be looking at some of the trial data part way through the trial. We may decide to drop one of the treatment groups if the data suggests the

treatment is unlikely to be beneficial or we may stop the trial early if the data suggests that none of the treatment groups are likely to be beneficial. This will

not affect your involvement in the trial at all. In both cases, the trial will be able to address the research questions.

**Will my data be kept confidential?**

If you decide to take part, you will be given an identification number for the trial, and all information collected about you for the trial will be linked to that number. This means only the people treating you, or who need to contact you, will have access to your personal information. The clinical research team or the central study team at The University of Sheffield may need to contact you to organise couriering of the TVNS device, arrange appointments or complete the trial questionnaires with you. If you wish to receive study newsletters and be informed of the study results we will collect your contact details and preferred method of contact, the central study team will use these details to send you this information. If you provide a mobile phone number we will use this to send automated text messages reminding you to complete your rehabilitation therapy and to attend your follow up appointments. You will receive text message reminders even if you have not selected mobile phone as your preferred method of contact. The central study team will have access to your consent form for monitoring purposes. The outcomes of this research will be published externally in a journal, on a website or via a conference presentation; however, you will not be identifiable from the published results. This means no one would be able to work out that you took part in the trial from the reports we write about it.

Your personal details will be kept strictly confidential.

**How will we use information about you?**
Sheffield Teaching Hospitals NHS Foundation Trust (STH NHSFT) is the sponsor that is leading this trial and will act as the data controller. This means that they are responsible for looking after your information and using it properly. The trial is managed by the Clinical Trials Research Unit (CTRU) in the School of Health and Related Research at The University of Sheffield.

Together STH NHSFT and the CTRU will need to use information from you and your medical records for this trial. This information will include your consent to take part in the trial, NHS number (or equivalent), name, contact details and date of birth. STH NHSFT and the CTRU will use this information to do the research or to check your records to make sure that the research is being done properly. Members of the research team who do not need to know who you are will not be able to see your name or contact details. Your data will have a code number instead.

We will inform your GP that you are taking part in this trial. We will keep all information about you safe and secure.

Sheffield Teaching Hospitals NHS Foundation Trust will collect information from you and your medical records for this research trial in accordance with instructions from Clinical Trials Research Unit at The University of Sheffield.

STH NHSFT, the CTRU and Sheffield Teaching Hospitals NHS Foundation Trust will keep your data securely for 15 years after the end of the trial.

**What are your choices about how your information is used?**
You can stop being part of the trial at any time, without giving a reason, but we will keep information about you that we already have.

- We need to manage the information that we collect in specific ways for the research to be reliable. This means that we won’t be able to let you see or change the data we hold about you.
- If you agree to take part in this trial, you may have the option to take part in future research using your data saved from this trial.
- The anonymised data may be used to support other research in the future, and may be shared with other researchers for comparison studies

**Where can you find out more about how your information is used?**
You can find out more about how we use your information here <https://www.sheffieldclinicalresearch.org/for-patients-public/how-is-your-information-handled-in-research/>

If you wish to raise a complaint on how we have handled your personal data, you can contact our Data Protection Officer who will investigate the matter. If you are not satisfied with our response or believe we are processing your personal data in a way that is not lawful you can complain to the Information Commissioner’s Office (ICO). The Sponsor’s Data Protection Officer is Michael Maginnis and you can contact them by phone (0114 2265153) or email (sth.infogov@nhs.net).

# Safeguarding concerns

If you are dissatisfied with any aspect of the research and wish to make a complaint, please contact [triceps@sheffield.ac.uk](mailto:triceps@sheffield.ac.uk) in the first instance. If you feel your complaint has not been handled in a satisfactory way you can contact the University of Sheffield Clinical Trials Research Unit Director, Professor Cindy Cooper ([c.l.cooper@sheffield.ac.uk](mailto:c.l.cooper@sheffield.ac.uk)). If the complaint relates to how your personal data has been handled, you can find information about how to raise a complaint in the University’s Privacy Notice: [*https://www.sheffield.ac.uk/govern/data-protection/privacy/general*](https://www.sheffield.ac.uk/govern/data-protection/privacy/general).

You are free to contact either STH NHSFT or The University of Sheffield to address any issues with how your data has been handled.

If you wish to make a report of a concern or incident relating to potential exploitation, abuse or harm resulting from your involvement in this project, please contact the project’s Designated Safeguarding Contact, through your local Patient Advice and Liaison Service (PALS) team 0114 271 2400 or email [sth.pals@nhs.net](mailto:sth.pals@nhs.net) If the concern or incident relates to the Designated Safeguarding Contact, or if you feel a report you have made to this Contact has not been handled in a satisfactory way, please contact the Dean of the School of Health and Related Research at The University of Sheffield, Professor Mark Strong ([m.strong@sheffield.ac.uk](mailto:m.strong@sheffield.ac.uk)) and/or the University’s Research Ethics & Integrity Manager (Lindsay Unwin; [l.v.unwin@sheffield.ac.uk](mailto:l.v.unwin@sheffield.ac.uk)).

**What will happen with the results of the research trial?**

The findings from this trial will be published in scientific journals and presented at scientific meetings. The findings will also be made available to patients through patient organisations, health information websites that are open to the public and the media where possible and appropriate. The trial website triceps-trial.com will publish a summary of the results following completion of the trial.

**What if there is a problem?**

If you have a concern about any aspect of this trial, you should ask to speak to the researchers who will do their best to answer your questions. The researchers contact details are at the end of this information sheet. If you wish to seek advice or reassurance about your own health, then contact your GP.

If you remain unhappy and wish to complain formally, you can do this by contacting the local NHS Patient Services Team:

Address: Patient Services Team, Sheffield Teaching Hospital NHS Foundation Trust, Royal Hallamshire Hospital, Glossop Road, Sheffield, S10 2JF

Telephone: 0114 271 2400

Email: [sth.pals@nhs.net](mailto:sth.pals@nhs.net)

**Who is organising and funding the research?**

The project is being carried out by a team of researchers from at the University of Sheffield (School of Health and Related Research and Sheffield Institute for Translational Neuroscience) and Sheffield Teaching Hospital NHS Foundation Trust. This trial is funded by the National Institute for Health Research Efficacy and Mechanism programme (project ref NIHR133169).

**Who has ethically reviewed the trial?**

All research in the NHS is looked at by an independent group of people, called a Research Ethics Committee to protect your safety, rights, wellbeing and dignity. This trial has been reviewed and given favourable opinion by East of England – Cambridge Central Research Ethics Committee (Ref: 22/EE/0209).

**Thank you for taking time to read this information sheet, we hope that it has been helpful in enabling you to decide if you would like to take part in the TRICEPS trial. This information sheet is for you to keep.**

For further information or if you have any questions, please find the research team’s contact details below:

Local Contact Details:

STH TRICEPS team email: Sth.triceps-studyteam@nhs.net

Stroke nurse phone number: 0114 2713749

Central Office Contact Details: [triceps@sheffield.ac.uk](mailto:triceps@sheffield.ac.uk)
