# Supplementary material for: TRanscutaneous lImb reCovEry Post-Stroke (TRICEPS): study protocol for a randomised, controlled, multiarm, multistage adaptive design trial
Source: BMJ Open. 2025 Mar 26;15(3):e092520. doi: 10.1136/bmjopen-2024-092520 (PMC11950934; doi:10.1136/bmjopen-2024-092520)
Supplement: online supplemental file 3 [file bmjopen-15-3-s003.doc]

**Participant Consent Form**

**TR**ranscutaneous l**I**mb re**C**ov**E**ry **P**ost-**S**troke **(TRICEPS)**

**Participant Identification Number for this trial: S /**

**MANDATORY RESPONSES**

Please initial box

1. I confirm that I have read the information sheet dated 17May2024 (Version 5.0) for the above study. I have had the opportunity to consider the information, ask questions and have had these answered satisfactorily.
2. I understand that my participation is voluntary and that I am free to withdraw at any time without giving any reason, without my medical care or legal rights being affected.
3. I understand that relevant sections of my medical notes and data collected during the study may be looked at by individuals from the Sheffield Clinical Trials Research Unit, from regulatory authorities or from the NHS Trust, where it is relevant to my taking part in this research. I give permission for these individuals to have access to my records.
4. I agree to my General Practitioner being informed of my participation in the study. I also agree to my GP being contacted, with my agreement, if the study team have any concerns about my health.
5. I understand that information collected by the research team, including a copy of this signed consent form, can be sent to and stored at the Sheffield Clinical Trials Research Unit for the purposes of monitoring and auditing.
6. I agree to return the TVNS device to the research after I have completed my 12 week rehabilitation therapy. My address may be provided to the central trial team to arrange
   a courier if required.
7. I agree that I will only use the mobile phone provided by the research team for the purposes of the trial.
8. Persons of child bearing potential only: I agree to carry out a pregnancy test to

Yes

N/A

confirm that I am not pregnant.

1. I agree to take part in the above study.

**OPTIONAL RESPONSES** Please initial the relevant box

Yes

No

1. I agree that data collected about me without personal identifiers may be used to support other research in the future and may be shared with other researchers
   for comparison studies; and I give my permission for this.

Yes

No

11. I would like to receive information, such as newsletters, about this research and understand that the central research team will have access to my contact details to send me such information.

Yes

No

12. I would like to be informed about the overall results of the TRICEPS trial and understand that the central research team will have access to my contact details to send me this information.

Yes

No

13. I agree that researchers can contact me regarding participation in other research.
I understand I may be contacted via post or email and be provided with further information and given the opportunity to decide whether or not to participate.

Name of Participant Date Signature

Name of Person Date Signature

requesting consent

**Interpreter / independent witness statement (if applicable)*** I have explained the above information to the participant to the best of my ability in a way which I believe the participant has understood.

Name of Interpreter/independent witness Date Signature

*If participant is physically unable to sign and date to confirm consent, an independent witness can do this on their behalf.  In such cases the participant should mark the consent form and the witness should sign to confirm the participant has given consent to take part in the study.

**If an interpreter / independent witness is required (please initial):**

I confirm that the interpreter / independent witness is NOT a member of the TRICEPS research team
