# Supplementary material for: TRanscutaneous lImb reCovEry Post-Stroke (TRICEPS): study protocol for a randomised, controlled, multiarm, multistage adaptive design trial
Source: BMJ Open. 2025 Mar 26;15(3):e092520. doi: 10.1136/bmjopen-2024-092520 (PMC11950934; doi:10.1136/bmjopen-2024-092520)
Supplement: online supplemental file 4 [file bmjopen-15-3-s004.docx]

**TRICEPS Mechanistic Sub-Study Participant Information Sheet**

Study Title: **TR**anscutaneous l**I**mb re**C**ov**E**ry **P**ost**-S**troke **(TRICEPS)**

Mechanistic Sub-Study

*As a part of the TRICEPS trial, you are invited to participate in a sub-study. Unfortunately, the sub-study is only available for patients within the Sheffield area or able to travel to Sheffield.*

*Before you decide whether to take part it is important that you understand why the research is being done and what it will involve. Please take time to read the following information sheet carefully and discuss it with friends, or relatives, if you wish. Please ask us if there is anything that is not clear to you or if you would like more information.*

**What is the purpose of this sub-study?**

The sub-study explores whether TVNS (a device that stimulates a nerve called the vagus nerve through the ear) alongside rehabilitation therapy produces changes in the brain. We will measure this using Magnetic Resonance Imaging (MRI). The changes that we will look at are brain plasticity (whether it can be ‘rewired’ to function in a different way), brain blood flow and energy usage. This will then be compared to what happens with rehabilitation therapy alone, without TVNS treatment. This comparison will be done with participants using the TVNS device and participants using the sham TVNS device (produces very low vagus nerve stimulation). We will look at whether these changes are linked to improvements in arm weakness in people who have had a stroke.

If we can understand these brain changes, it could help us predict which patients are most likely to respond to TVNS therapy. This could also help us improve TVNS treatments and allow the treatment to be personalised to patients.

TVNS may also have effects on the level of signalling proteins that reduce inflammation and increase blood flow in the brain. By taking blood samples before and after TVNS therapy, this could help us show how TVNS works and

the effects it has on the body. This may also help show which participants are responding to TVNS.

**Why have I been invited to take part in the sub-study?**

You have been invited to take part as you are taking part in the TRICEPS trial. You are also located in the Sheffield area or able to travel to Sheffield.

Do I have to take part?

No, you do not have to take part; it is up to you to decide. We will describe the sub-study throughout this information sheet, which you can keep. You do not have to decide today. If you do not wish to decide today, you can let us know at a later date using the research team’s contact details at the end of this information sheet. If we have not heard from you the research team will give you a call about a week after giving you this information sheet to check if you would like to take part. If you do agree to take part, you are free to withdraw at any time, without giving a reason.

The sub-study is optional. You can take part in the main part of the trial, even if you choose not to take part in the sub-study.

**What would taking part involve?**

The sub-study involves two MRI scans. Some participants may be asked if they would be happy to have a Positron Emission Tomography (PET) scan at the same time. This is called a PET-MRI scan. The PET scan is optional. If you are invited to have a PET scan you can choose to have both the MRI and PET scan or just the MRI scan.

We will ask you to sign a consent form to confirm which scans you agree to have. You will know which scans you are having before you attend your appointment.

A member of the research team will confirm that you are eligible to take part in the sub-study. We will then make an appointment for you to attend the MRI department at the Royal Hallamshire Hospital in Sheffield.

Your MRI (and PET, if applicable) appointment must take place before you start your TVNS and therapy treatment.

**What will happen at my appointment?**

When you attend your appointment, a blood sample will be taken by a trained professional. This will be just like having a standard blood test. This blood sample will be frozen and stored in a secure laboratory in the University of Sheffield which is accessible to members of the research team. The frozen blood samples can then be analysed in the future – for example, to look at the levels of signalling proteins that affect inflammation and blood flow and to analyse whether TVNS changes these.

We will then go through a checklist of questions with you. MRI scanning is generally very safe but there are certain circumstances when it must be avoided so this checklist is to make sure it is safe for you. The checklist will include questions about metal objects attached to or inside your body (e.g. stents, shrapnel, plated fractures) or electronic devices (e.g. heart pace-maker). Many such items (most modern cardiac stents, artificial knees and hips, for instance) are designed to be MRI safe, but we do need to know about them. You will be seen by a member of staff in the MRI department before your scan and they will explain to you what will happen during the scan. You will also be able to ask questions before and after the scan takes place.

You will be asked to lie still on a bed in the scanner’s ‘tunnel’. The ‘tunnel’ is quite narrow so please let us know if you are likely to have feelings of being enclosed or claustrophobia before your scan. During the scan you will hear a loud noise made by the MRI scanner as it is working. You will be given earplugs to reduce this noise. The technician performing the MRI scan will talk to you during the scan to check that you are comfortable. The scan can be stopped at any point, but in most cases, it will take about 1 hour.

****PET-MRI scan only***

If you agree to have a PET-MRI scan you will be asked not to eat or drink for 6 hours before your appointment.

Female participants, who could become pregnant, may be asked to have a pregnancy test before the scan to exclude the possibility of pregnancy. Following your scan the pregnancy test will be destroyed in line with usual practice at the hospital.

You will have an injection of a radioactive ‘tracer’ or ‘dye’. This tracer goes to different parts of the body, the PET scanner detects where the tracer has built up and will create an image. The injected radioactivity fades away naturally over a few hours and you can leave the scanning centre as soon as the scan is finished. The PET scan will be taken at the same time as the MRI scan.

After the scan you should drink plenty of water to help flush out the tracer from your kidneys. You should avoid contact with pregnant women and young children for about 8 hours after the scan while the radioactive tracer is still in your system.

**What happens next?**

You will then have a follow-up scan about 3 months later once you have finished your TVNS and therapy treatment. If you had a PET scan at your first appointment you will also have one at your follow up appointment. We will also take another blood sample.

Occasionally, brain scanning may find a medical health problem which we didn’t expect. If this happens, we will report this to your GP who will arrange any further investigations or care that you might need.

**What are the possible benefits of taking part?**

The sub-study does not involve any treatment so there is no direct benefit to your health from taking part. By taking part you will be making a significant contribution to medical knowledge about stroke recovery.

**What are the possible disadvantages or risks of taking part?**

There are some possible disadvantages to having an MRI scan, as some people can find being in the scanner uncomfortable or unpleasant (please see above “**What will happen at my appointment?**”). It is important to point out though

that most people experience little or no problem at all when they are having their MRI scan.

A PET scan is a safe test for most people. But like all medical tests it has some risks. The MRI team will discuss these with you at your appointment and you will be able to ask questions.

The radioactive tracer (or “dye”) used in PET scans emits ionising radiation. This radiation is what is used to form the PET image, but it can also cause cell damage that may, after many years or decades, turn cancerous. We are all at risk of developing cancer during our lifetime. The normal risk is that this will happen to about 1 in 2 people at some point in their life. This procedure will only add a small chance of this happening to you (less than 1 in 2,500). The doctors caring for you consider that the benefits to society and future patient groups outweigh any risk from the radiation.

Some people can have an allergic reaction to the radioactive tracer but this is very rare. If you start to feel unwell, please tell the MRI team immediately.

Attending the appointments will take around 1 and a half hours of your time for the MRI only appointment and around 3 hours of your time for the PET-MRI scan appointment.

What will happen if I do not want to carry on with the sub-study?

You can withdraw from the sub-study at any time without giving any reason. We will keep your data until the point that you withdraw, and we will not collect any new information from you.

What if new information becomes available?

Sometimes during the course of a research study, new information becomes available about the intervention that is being studied. If this happens, the research team will tell you about it and discuss with you whether you want to continue in the study. If you decide to continue in the sub-study, you will be asked to sign an updated consent form.

In some circumstances, on receiving new information the researcher might consider it to be in your best interests to withdraw you from the study. The researcher will explain the reasons.

**How will we use information about you?**
Sheffield Teaching Hospitals NHS Foundation Trust (STH NHSFT) is the sponsor that is leading this trial and will act as the data controller. This means that they are responsible for looking after your information and using it properly. The trial is managed by the Clinical Trials Research Unit (CTRU) in the School of Health and Related Research at The University of Sheffield.

Together STH NHSFT and the CTRU will need to use information from you and your medical records for this trial. This information will include your consent to take part in the trial, NHS number (or equivalent), name, contact details and date of birth. STH NHSFT and the CTRU will use this information to do the research or to check your records to make sure that the research is being done properly. Members of the research team who do not need to know who you are will not be able to see your name or contact details. Your data will have a code number instead.

We will inform your GP that you are taking part in this trial.

We will keep all information about you safe and secure.

[E*nter site name*] will collect information from you and your medical records for this research trial in accordance with instructions from Clinical Trials Research Unit at The University of Sheffield.

STH NHSFT, the CTRU and [*enter site name*] will keep your data securely for 15 years after the end of the trial.

No one would be able to work out that you took part in the trial from the reports we write about it.

**What are your choices about how your information is used?**
You can stop being part of the trial at any time, without giving a reason, but we will keep information about you that we already have.

- We need to manage the information that we collect in specific ways for the research to be reliable. This means that we won’t be able to let you see or change the data we hold about you.
- If you agree to take part in this trial, you may have the option to take part in future research using your data saved from this trial.
- The anonymised data may be used to support other research in the future, and may be shared with other researchers for comparison studies

**Where can you find out more about how your information is used?**
You can find out more about how we use your information here <https://www.sheffieldclinicalresearch.org/for-patients-public/how-is-your-information-handled-in-research/>

If you wish to raise a complaint on how we have handled your personal data, you can contact our Data Protection Officer who will investigate the matter. If you are not satisfied with our response or believe we are processing your personal data in a way that is not lawful you can complain to the Information Commissioner’s Office (ICO). The Sponsor’s Data Protection Officer is Michael Maginnis and you can contact them by phone (0114 2265153) or email (sth.infogov@nhs.net).

# **Safeguarding concerns**

If you are dissatisfied with any aspect of the research and wish to make a complaint, please contact [triceps@sheffield.ac.uk](mailto:triceps@sheffield.ac.uk) in the first instance. If you feel your complaint has not been handled in a satisfactory way you can contact the University of Sheffield Clinical Trials Research Unit Director, Professor Cindy Cooper ([c.l.cooper@sheffield.ac.uk](mailto:c.l.cooper@sheffield.ac.uk)) If the complaint relates to how your personal data has been handled, you can find information about how to raise a complaint in the University’s Privacy Notice: [*https://www.sheffield.ac.uk/govern/data-protection/privacy/general*](https://www.sheffield.ac.uk/govern/data-protection/privacy/general).

You are free to contact either STH NHSFT or The University of Sheffield to address any issues with how your data has been handled.

If you wish to make a report of a concern or incident relating to potential exploitation, abuse or harm resulting from your involvement in this project, please contact the project’s Designated Safeguarding Contact, through your local Patient Advice and Liaison Service (PALS) team [Insert site-specific detail]. If the concern or incident relates to the Designated Safeguarding Contact, or if you feel a report you have made to this Contact has not been handled in a satisfactory way, please contact the Dean of the School of Health and Related Research at The University of Sheffield, Professor Mark Strong ([m.strong@sheffield.ac.uk](mailto:m.strong@sheffield.ac.uk)) and/or the University’s Research Ethics & Integrity Manager (Lindsay Unwin; [*l.v.unwin@sheffield.ac.uk*](mailto:l.v.unwin@sheffield.ac.uk)).

What will happen with the results of the research trial?

The findings from this trial will be published in scientific journals and presented at scientific meetings. The findings will also be made available to patients through patient organisations, health information websites that are open to the public and the media where possible and appropriate. The trial website triceps-trial.com will publish a summary of the results following completion of the trial.

What if there is a problem?

If you have a concern about any aspect of this trial, you should ask to speak to the researchers who will do their best to answer your questions. The researchers contact details are at the end of this information sheet.

If you remain unhappy and wish to complain formally, you can do this by contacting the local NHS Patient Services Team:

Address: <insert address>

Telephone: <insert phone number>

Email: <insert email>

Who is organising and funding the research?

The project is being carried out by a team of researchers from at the University of Sheffield School of Health and Related Research and Sheffield Institute of Translational Neuroscience) and Sheffield Teaching Hospital NHS Foundation Trust. This trial is funded by the National Institute for Health Research Efficacy and Mechanism programme (project ref NIHR133169).

Who has ethically reviewed the trial?

All research in the NHS is looked at by an independent group of people, called a Research Ethics Committee to protect your safety, rights, wellbeing and dignity. This trial has been reviewed and given favourable opinion by East of England – Cambridge Central Research Ethics Committee (Ref: 22/EE/0209).

**Thank you for taking the time to read this information sheet. We hope that it has helped you to decide if you would like to take part in the TRICEPS sub-study. This information sheet is for you to keep.**

For further information or if you have any questions, please find the research team’s contact details below:

Local Contact Details:

[Add local NHS Trust details]

Central Office Contact Details: [triceps@sheffield.ac.uk](mailto:triceps@sheffield.ac.uk)
