# Supplementary material for: TRanscutaneous lImb reCovEry Post-Stroke (TRICEPS): study protocol for a randomised, controlled, multiarm, multistage adaptive design trial
Source: BMJ Open. 2025 Mar 26;15(3):e092520. doi: 10.1136/bmjopen-2024-092520 (PMC11950934; doi:10.1136/bmjopen-2024-092520)
Supplement: online supplemental file 5 [file bmjopen-15-3-s005.doc]

**Mechanistic Sub-Study Participant Consent Form**

**TR**ranscutaneous l**I**mb re**C**ov**E**ry **P**ost-**S**troke **(TRICEPS)**

Mechanistic Sub-Study

**Participant Identification Number for this trial: S /**

**MANDATORY RESPONSES**

Please initial box

1. I confirm that I have read the information sheet dated.................... (version............) for the above study. I have had the opportunity to consider the information, ask questions and have had these answered satisfactorily.
2. I understand that I will have the opportunity to ask further questions about the MRI scan itself before this takes place.
3. I understand that my participation is voluntary and that I am free to withdraw at any time
   without giving any reason, without my medical care or legal rights being affected.
4. I understand that relevant sections of my medical notes and data collected during the study may be looked at by individuals from the Sheffield Clinical Trials Research Unit, from regulatory authorities or from the NHS Trust, where it is relevant to my taking part in this research. I give permission for these individuals to have access to my records.
5. I agree to my General Practitioner being informed of my participation in the study and relevant information about me being exchanged between my GP and the research team.
6. I understand that anonymous images from my MRI scan may be used for training, teaching and/or publication in medical journals.
7. I agree to have a blood sample taken and for it to be stored and used for future research relevant to this project.
8. I agree to have a follow up MRI approximately 3 months after my first scan.

Please initial box

1. I understand that information collected by the research team, including a copy of this signed consent form, can be sent to and stored at the Sheffield Clinical Trials Research Unit for the purposes of monitoring and auditing.
2. I agree to take part in the above study.

**OPTIONAL RESPONSES** Please initial the relevant box

*NOTE: once we reach maximum recruitment for the optional PET-MRI items 11 and 12 will be removed from the consent form*

Yes

No

1. I agree to have a PET-MRI during the same appointment for the above MRI scan

Yes

No

1. I understand that I will be injected with a radiotracer for the purpose of the PET-MRI during the same appointment for the above MRI scan (required for option 11).

Yes

No

1. I understand that the information that collected about me may be used to support other research in the future and may be shared anonymously with other researchers and I give my permission for this.

Yes

No

1. I agree that researchers can contact me regarding participation in other research.
   I understand I will be provided with further information and given the opportunity
   to decide whether or not to participate.

Name of Participant Date Signature

Name of Person Date Signature

requesting consent

**Interpreter / independent witness statement (if applicable)*** I have explained the above information to the participant to the best of my ability in a way which I believe the participant has understood.

Name of Interpreter/ Date Signature

Independent Witness

*If participant is physically unable to sign and date to confirm consent, an independent witness can do this on their behalf.  In such cases the participant should mark the consent form and the witness should sign to confirm the participant has given consent to take part in the study.

**If an interpreter / independent witness is required (please initial):**

I confirm that the interpreter / independent witness is NOT a member of the TRICEPS research team
