# Supplementary material for: TRanscutaneous lImb reCovEry Post-Stroke (TRICEPS): study protocol for a randomised, controlled, multiarm, multistage adaptive design trial
Source: BMJ Open. 2025 Mar 26;15(3):e092520. doi: 10.1136/bmjopen-2024-092520 (PMC11950934; doi:10.1136/bmjopen-2024-092520)
Supplement: online supplemental file 6 [file bmjopen-15-3-s006.docx]

| Data category | Information |
| --- | --- |
| Primary registry and trial identifying number | ISRCTN ISRCTN20221867 |
| Date of registration in primary registry | 11 June, 2010 |
| Secondary identifying numbers | N/A |
| Source(s) of monetary or material support | National Institute for Health and Care Research |
| Primary sponsor | Sheffield Teaching Hospitals NHS Foundation Trust |
| Secondary sponsor(s) | N/A |
| Contact for public queries | [triceps@sheffield.ac.uk](mailto:triceps@sheffield.ac.uk) |
| Contact for scientific queries | triceps@sheffield.ac.uk |
| Public title | A trial investigating the effectiveness of vagus nerve stimulation during rehabilitation therapy in patients with a weak arm following a stroke |
| Scientific title | An efficacy and mechanism evaluation of transcutaneous vagal nerve stimulation for upper limb recovery post-stroke – a randomised, controlled, multi-arm, multi-stage, adaptive design trial |
| Countries of recruitment | United Kingdom |
| Health condition(s) or problem(s) studied | Chronic stroke, arm weakness |
| Intervention(s) | Active comparator: Active transcutaneous auricular vagus nerve stimulation paired with upper limb rehabilitation. |
|  | Placebo comparator: Sham (low-amplitude) transcutaneous auricular vagus nerve stimulation paired with upper limb rehabilitation |
| Key inclusion and exclusion criteria | Ages eligible for study: ≥18 years Sexes eligible for study: both Accepts healthy volunteers: no |
|  | Inclusion criteria: adult patient (≥ 18 years); anterior circulation ischaemic stroke between 6 months and 10 years previously; baseline Upper Limb Fugl-Meyer total motor score of 20 – 50 (inclusive); at least 10 degrees of active wrist extension, thumb abduction/extension and extension in at least 2 additional digits; able to participate in rehabilitation therapy. |
|  | Exclusion criteria: other significant impairment of the upper limb (e.g. frozen shoulder), health conditions that prevent engagement with rehabilitation therapy (e.g. advanced dementia) |
| Study type | Interventional |
|  | Allocation: randomised controlled trial |
|  | Primary purpose: treatment |
|  | Phase – not applicable |
| Date of first enrolment | November 2023 |
| Target sample size | 243 |
| Recruitment status | Recruiting |
| Primary outcome(s) | Upper limb motor function assessed using the Upper Limb Fugl-Meyer total motor score at 3 months from the start of treatment. |
| Key secondary outcomes | Upper Limb Fugl-Meyer total score, Wolf Motor Function test, Modified Rankin Scale, Nottingham Extended Activities of Daily Living Scale, Stroke-Specific Quality of Life scale. |

Appendix Table 1. Trial Registration Data Set
