# Supplementary material for: TRanscutaneous lImb reCovEry Post-Stroke (TRICEPS): study protocol for a randomised, controlled, multiarm, multistage adaptive design trial
Source: BMJ Open. 2025 Mar 26;15(3):e092520. doi: 10.1136/bmjopen-2024-092520 (PMC11950934; doi:10.1136/bmjopen-2024-092520)
Supplement: online supplemental file 7 [file bmjopen-15-3-s007.docx]

**Supplemental Table 2. Overview of the schedule of procedures and outcome measure assessments**

|  | | **Visit** | | | | | |
| --- | --- | --- | --- | --- | --- | --- | --- |
|  |  | **Telephone / Video screening** | **Baseline (enrolment visit)*** | **Substudy visit** | **Treatment contact reviews** | **3-month follow-up** | **6-month follow-up** |
| **Mode of contact** | | Remote | Face-to-face | Face-to-face | Remote | Face-to-face | Face-to-face |
| **Timescale** | | Pre-enrolment | Week 0 | Prior to commencing treatment (max 4 weeks) | Weeks 1, 3, 5, 7 and 9 | 91 days (86 – 105 days) | 182 days (177 – 196 days) |
| **Activity** | |  |  |  |  |  |  |
| Eligibility | Eligibility screening | ✔ |  |  |  |  |  |
|  | Informed consent |  | ✔ |  |  |  |  |
|  | Eligibility form |  | ✔ |  |  |  |  |
| Outcome measures | ULFM |  | ✔ |  |  | ✔ | ✔ |
|  | WMFT |  | ✔ |  |  | ✔ | ✔ |
|  | mNIHSS |  | ✔ |  |  | ✔ | ✔ |
|  | mRS |  | ✔ |  |  | ✔ | ✔ |
|  | MRC Power |  | ✔ |  |  | ✔ | ✔ |
|  | MAS |  | ✔ |  |  | ✔ | ✔ |
|  | NEADL |  | ✔ |  |  | ✔ | ✔ |
|  | SS-QOL |  | ✔ |  |  | ✔ | ✔ |
|  | GAD-7 |  | ✔ |  |  | ✔ | ✔ |
|  | PHQ-9 |  | ✔ |  |  | ✔ | ✔ |
|  | NFI-Stroke |  | ✔ |  |  | ✔ | ✔ |
|  | VAS |  |  |  |  |  |  |
| Randomisation |  |  | ✔ |  |  |  |  |
| Device and therapy training |  |  | ✔ |  |  |  |  |
| Adverse event monitoring |  |  |  | ✔ | ✔ | ✔ | ✔ |
| Substudy-specific | MRI scan +/- FDG-PET scan |  |  | ✔ |  | ✔ |  |
|  | Blood sample |  |  | ✔ |  | ✔ |  |

**Key:** ULFM, Upper Limb Fugl Meyer; WMFT, Wolf Motor Function Test; mNIHSS, Modified National Institutes of Health Stroke Scale; MRC Power Score, Medical Research Council Muscle Power Scores (upper limb); MAS, Modified Ashworth Scale; NEADL, Nottingham Extended Activities of Daily Living; SS-QOL, Stroke Specific Quality of Life Scale; GAD-7, Generalised Anxiety Disorder Questionnaire, PHQ-9, Patient Health Questionnaire 9, NFI-Stroke, Neurological Fatigue Index for Stroke, VAS, Visual Analogue Scale for pain.
 ***May be split over 2 visits.**
