# Supplementary material for: TRanscutaneous lImb reCovEry Post-Stroke (TRICEPS): study protocol for a randomised, controlled, multiarm, multistage adaptive design trial
Source: BMJ Open. 2025 Mar 26;15(3):e092520. doi: 10.1136/bmjopen-2024-092520 (PMC11950934; doi:10.1136/bmjopen-2024-092520)
Supplement: Supplementary file 8 [file bmjopen-2024-092520.reviewer_comments.pdf]

PEER REVIEW HISTORY

BMJ Open publishes all reviews undertaken for accepted manuscripts. Reviewers are asked to complete a checklist review form and are provided with free text boxes to elaborate on their assessment. These free text comments are reproduced below.

ARTICLE DETAILS

Title (Provisional)

TRanscutaneous lImb reCovEry Post-Stroke (TRICEPS) – study protocol for a randomised, controlled, multi-arm, multi-stage adaptive design trial.

Authors

Baig, Sheharyar S; Mooney, Cara; McKendrick, Kirsty; Duffy, Kate E. M.; Ali, Ali N.; Redgrave, Jessica N.; Herbert, Esther; Waterhouse, Simon; Su, Li; Drummond, Avril; Dawson, Jesse; Dimairo, Munyaradzi; Biggs, Katie; Cooper, Cindy; Majid, Arshad

---

VERSION 1 - REVIEW

**Reviewer**	**1**
**Name**	**Blbas, Hazhar Talaat Abubaker**
**Affiliation**	**Salahaddin University - Erbil, Applied Statistics**
**Date**	**24-Sep-2024**
**COI**	**None**

There is no objective of study and hypothesis of study

There are no results

This paper is too long

**Reviewer**	**2**
**Name**	**Khan, Fayaz**
**Affiliation**	**King Abdulaziz University, Physical Therapy**
**Date**	**27-Dec-2024**
**COI**	**None**

The authors have made substantial efforts for designing the study protocol titled "TRanscutaneous lImb reCovEry Post-Stroke (TRICEPS) – study protocol for a randomised, controlled, multi-arm, multi-stage adaptive design trial". tANVS is a novel non invasive brain stimulation technique which was recently introduced in rehabilitation of patients with

stroke. Hence, a clinical trial with a large sample is needed to prove its efficacy and tranformation to clinical practice.

Few points needs clarification:

1. In the patient population: Does the investigators have plans to subcategorise/stratify the anterior circulation stroke to middle cerebral artery stroke and anterior cerebral artery stroke.

2. Justification needed for long duration for inclusion. As the potential for recovery is different when the duration of stroke is too long.

3. In the sample size justification: The dropout rate of 6% mentioned here is with a previous reference ?

4. In adverse events:

It would be better to monitor heart rate and blood pressure after the tVNS application or at regular intervals to maintain the safety for patients; some studies have reported an average change in the blood pressure of 2 to 5 points after the tVNS application.

**Reviewer**	**3**
**Name**	**Gao, Qiang**
**Affiliation**	**Sichuan University**
**Date**	**01-Jan-2025**
**COI**	**None**

The study protocol for the TRICEPS trial presents a well-structured and comprehensive approach to investigating the effects of transcutaneous vagus nerve stimulation (tVNS) on arm recovery in chronic stroke. However, there are several areas that require attention before the manuscript can be considered for publication.

Major Concerns:

1. In the Introduction section, the authors explain the mechanisms of Vagus Nerve Stimulation (VNS), but they do not provide sufficient discussion on the relative advantages of tVNS compared to VNS, nor do they address whether there are any differences in treatment outcomes between the two. A more thorough exploration of these aspects would strengthen the rationale for using tVNS in this trial.

2. In the introduction section, the authors summarize the mechanisms through which VNS improves upper limb function in stroke patients, but they do not provide any relevant information on the current state of research on tVNS. The gap in research and the logical flow are not fully addressed. A more thorough discussion of the current literature on tVNS and the identified research gap would strengthen the rationale for this study.

3. It is important to clarify the duration of tVNS across the different groups. While Group C receives additional tVNS stimulation for 1-8 hours daily during activities of daily living, it is stated that Groups A and B do not receive this additional stimulation. How does the study control for this difference in stimulation duration between the groups? A more detailed explanation of how this variable is managed would strengthen the study's design and control over potential confounding factors.

4. Since Group C receives additional tVNS stimulation during daily activities (1-8 hours per day), while Groups A and B do not, how does the study maintain blinding and control for this variation in treatment intensity? It is crucial to ensure that participants and investigators are unaware of the treatment group allocation to prevent bias.More detailed information on how blinding is maintained, particularly with regard to the additional stimulation in Group C, would help clarify the study's methodology.

5. It would be beneficial to include more detailed information on the processing and analysis of functional MRI (fMRI) data. Specifically, the methodology for preprocessing fMRI data, including steps such as motion correction, normalization, and statistical modeling, should be clearly outlined. Additionally, the analysis pipeline for assessing cortical plasticity, cerebral blood flow, and brain oxygen metabolism should be described in greater detail, particularly regarding the specific software and statistical approaches used.

Minor Concerns:

1. Regarding the inclusion and exclusion criteria, such as the Baseline Upper Limb Fugl Meyer total motor score of 20-50 indicating moderate to severe arm motor impairment, the authors should provide supporting references for these criteria. Citing relevant studies or guidelines would strengthen the rationale behind these thresholds.

2. It is strongly recommended to include a flow diagram outlining the study design. A clear schematic representation of the participant timeline, including enrollment, interventions

(such as tVNS stimulation protocols), and assessments, would greatly enhance the clarity of the study's methodology.

3. There are several grammatical errors and formatting inconsistencies throughout the manuscript. A thorough proofreading is recommended to improve the overall readability and professionalism of the document.

VERSION 1 - AUTHOR RESPONSE

Reviewer 1	There is no objective of study and hypothesis of study<br>There are no results<br>This paper is too long	Thank you to Reviewer 1 for reading the manuscript. There appears to have been some misinterpretation of the manuscript.<br><br>As a protocol of an ongoing study there are no results as of yet.<br><br>The manuscript length fits within the guidelines of the journal.<br><br>We would argue that there is a clear study objective and hypothesis. We appreciate this may not have been made as clear as possible so have included a dedicated section on study aims and hypotheses.	Page 5
Reviewer 2	In the patient population: Does the investigators have plans to subcategorise/stratify the anterior circulation stroke to middle cerebral artery stroke and anterior cerebral artery stroke.	Thank you. We expect that the total number of participants with a pure anterior cerebral artery stroke in this study will be low as this typically causes a leg monoparesis rather than arm involvement.<br><br>We will report on the proportion of participants with the presence or absence of cortical involvement in each trial arm (i.e. comparing the proportion of participants with non-lacunar vs lacunar anterior circulation stroke) in line with previous work in this area (VNS-REHAB study).	N/A

		This will be presented in the reported participant characteristics and/or in subsequent post-hoc analyses looking at predictors of treatment response.	
Reviewer 2	Justification needed for long duration for inclusion. As the potential for recovery is different when the duration of stroke is too long.	Thank you. This is an important point and one which was discussed extensively in the trial planning phase and with consultation with stroke survivors. The window for spontaneous biological recovery is largely within the first 6 months after stroke. In the longer term, significant clinical improvements in upper limb function can be made with intensive rehabilitation (doi: 10.1136/jnnp-2019-320441, doi:10.1016/j.apmr.2014.10.022). These do not appear to be limited by time since stroke. For instance, in the Queen Square Upper Limb Neurorehabilitation study the median (IQR) for time since stroke in months was 18 (12 – 51) with no restriction in time since stroke.<br><br>Whilst we appreciate some aspects of limb impairment can become more likely if untreated in the long term (e.g. contractures), the participants with the most severe impairment and restriction in movements are ineligible in our inclusion/exclusion criteria.<br><br>This study was codesigned with stroke survivors and advocates who felt that the programme was appropriate and manageable for people even up to 10 years post-stroke.	Page 7

		This discussion point is elaborated on in the revised manuscript.	
Reviewer 2	In the sample size justification: The dropout rate of 6% mentioned here is with a previous reference ?	Thank you for this point. One of the largest studies of upper limb rehabilitation in chronic stroke (The Queen Square Upper Limb Neurorehabilitation Programme) has a similar study population and reported a 5.8% dropout from their 6-month follow-up schedule. This has been included as a reference.<br>Please note that this dropout rate will be reviewed at an interim analysis and sample size updated accordingly as highlighted under "Sample size justification and interim analysis" section.	Page 10
Reviewer 2	In adverse events:<br>It would be better to monitor heart rate and blood pressure after the tVNS application or at regular intervals to maintain the safety for patients; some studies have reported an average change in the blood pressure of 2 to 5 points after the tVNS application.	Thank you for this point.<br><br>As the auricular branch of the vagus nerve is a purely afferent nerve does not have any direct efferent connections to cardiac control. A recent systematic review of studies of taVNS reports that in 484 212 min-days of stimulation, no adverse cardiac events were reported (https://doi.org/10.1038/s41598-022-25864-1). If symptoms suggestive of adverse cardiac events (e.g. palpitations, dizziness, pre-syncope) are reported by participants then these will be investigated accordingly. However, as a home -delivered intervention (60 sessions over 12 weeks), it is impractical to have serial heart rate and blood pressure assessments.<br><br>Further detail on the safety of	Page 11

		taVNS is now included in the manuscript for clarification.	
Reviewer 3	In the Introduction section, the authors explain the mechanisms of Vagus Nerve Stimulation (VNS), but they do not provide sufficient discussion on the relative advantages of tVNS compared to VNS, nor do they address whether there are any differences in treatment outcomes between the two. A more thorough exploration of these aspects would strengthen the rationale for using tVNS in this trial.	Thank you for this comment. As you mention, the potential advantages of tVNS over VNS are several-fold (cost, safety profile, scalability). These points are now discussed in the Introduction.<br><br>The treatment effects are difficult to compare as there is a very limited evidence base for tVNS in chronic stroke. The previous studies are harder to compare as they involve different therapy approaches (e.g. robotic rehabilitation) or include participants with subacute stroke. Nevertheless, our previous pilot study showed a mean increase of 10.1 points in the ULFM motor score. This detail has been included for context in the Introduction.	Page 4-5
Reviewer 3	In the introduction section, the authors summarize the mechanisms through which VNS improves upper limb function in stroke patients, but they do not provide any relevant information on the current state of research on tVNS. The gap in research and the logical flow are not fully addressed. A more thorough discussion of the current literature on tVNS and the identified research gap would strengthen the rationale for this study	Thank you for this suggestion. We have recently reviewed the current evidence base for tVNS in stroke and have separately reviewed international clinical trials registries for ongoing studies of tVNS to identify the key research gaps. The key areas from this recent study are now outlined in the introduction and these reviews are referenced.	Page 4-5
Reviewer 3	It is important to clarify the duration of tVNS across the different	The study team are keen to avoid the inclusion of information that could directly unblind	N/A

	groups. While Group C receives additional tVNS stimulation for 1-8 hours daily during activities of daily living, it is stated that Groups A and B do not receive this additional stimulation. How does the study control for this difference in stimulation duration between the groups? A more detailed explanation of how this variable is managed would strengthen the study's design and control over potential confounding factors.	participants. We have rewritten the section on Intervention to not specify doses of tVNS or duration of tVNS use. The final publication of the study results will include details of the stimulation parameters in all groups, its limitations and the overall treatment durations.	
Reviewer 3	Since Group C receives additional tVNS stimulation during daily activities (1-8 hours per day), while Groups A and B do not, how does	Participants in the trial are not made aware of the stimulation parameters in Group C (i.e. whether Group C receives either active or sham stimulation). All outcomes assessors are blinded	Page 8

	the study maintain blinding and control for this variation in treatment intensity? It is crucial to ensure that participants and investigators are unaware of the treatment group allocation to prevent bias. More detailed information on how blinding is maintained, particularly with regard to the additional stimulation in Group C, would help clarify the study's methodology.	to the group allocation of participants. Rehabilitation interventions and device set up are carried out by separate unblinded trial participants. Blinded assessors are advised to not discuss details of the device or rehabilitation elements of the programme with participants to avoid risk of unblinding. If assessors are unwillingly unblinded, they are asked to report this to the central study team and for a separate individual to carry out the assessment.<br><br>These details have been added to the revised manuscript.	
Reviewer 3	It would be beneficial to include more detailed information on the processing and analysis of functional MRI (fMRI) data. Specifically, the methodology for preprocessing fMRI data, including steps such as motion correction, normalization, and statistical modeling, should be clearly outlined. Additionally, the analysis pipeline for assessing cortical plasticity, cerebral blood flow, and brain oxygen metabolism should be described in greater detail, particularly regarding the specific software and statistical approaches used.	Thank you. We are carrying out several types of analysis using the MRI data. Sequences include T1, T2, T2 GRE, FLAIR, ASL, resting state fMRI, task fMRI and diffusion tensor imaging. The analysis will follow established methodology including the preprocessing steps listed. Given the complexity of working with imaging data from individuals with lesions and the variability of different metrics depending on severity of stroke and patient concordance with the imaging protocols, we cannot prespecify the overall analysis pipeline. This will require an iterative process for identifying the optimal signal to noise ratio from different software packages and may incorporate > 1 software in the preprocessing pipeline. Where these steps have been established, details are now included in the manuscript. Further detailed steps will be reported extensively in the	Page 12

		imaging publications resulting from this dataset.	
Reviewer 3	Regarding the inclusion and exclusion criteria, such as the Baseline Upper Limb Fugl Meyer total motor score of 20-50 indicating moderate to severe arm motor impairment, the authors should provide supporting references for these criteria. Citing relevant studies or guidelines would strengthen the rationale behind these thresholds.	Thank you. The criteria of FMA-UE 20-50 was chosen to be in line with comparable studies such as the VNS-REHAB trial. This reference is now included as suggested.<br><br>To elaborate further, for those scoring < 20, the study team felt that active rehabilitation with repetitive task practice involving purposeful hand movements would be limited thereby potentially limiting the potential benefits of this rehabilitation strategy on upper limb recovery. For scores > 50, the study team felt that the impairment is relatively less restrictive and that there were fewer impairments in flexor synergy. As such, there would be a potential ceiling effect from treatment.	Pages 6-7
Reviewer 3	It is strongly recommended to include a flow diagram outlining the study design. A clear schematic representation of the participant timeline, including enrolment, interventions (such as tVNS stimulation protocols), and assessments, would greatly enhance the clarity of the study's methodology.	Thank you. This has now been included as Figure 1.	Figure 1
Reviewer 3	There are several grammatical errors and formatting inconsistencies throughout the manuscript. A thorough	Thank you. These have been addressed with amendments to grammar and formatting throughout.	Throughout.

	proofreading is recommended to improve the overall readability and professionalism of the document.		
